# Analysis of Photothermal Conversion Behaviors in Graphene–Polymer Nanocomposites

**DOI:** 10.3390/polym18080968

**Published:** 2026-04-16

**Authors:** Haiyu Zhang, Runzhe Rao, Yan Feng, Zhou Fang, Xinyan Hu, Fang Li

**Affiliations:** 1 Green & Smart River-Sea-Going Ship, Cruise and Yacht Research Center, Wuhan University of Technology, Wuhan 430070, China; zhanghaiyu@whut.edu.cn (H.Z.);; 2 Hubei Key Laboratory of Theory and Application of Advanced Materials Mechanics, Wuhan University of Technology, Wuhan 430070, China

**Keywords:** graphene–polymer nanocomposites, photothermal conversion, interface effects, optical conductivity, near-infrared light

## Abstract

Due to its strong near-infrared (NIR) absorption and high thermal conductivity, graphene is considered an excellent nanophotothermal filler that can effectively improve the photothermal conversion performance of composites. In particular, graphene–polymer nanocomposites, new types of photothermal conversion materials, have broad application prospects in photothermal therapy, photothermal driving, and micro-/nanomachinery. Recent research results have shown that when the filling concentration of graphene nanosheets (GNSs) in the matrix reaches the percolation threshold, interface effects such as interface tunneling and Maxwell–Wagner–Sillars (MWS) polarization, the key factors affecting the photothermal conversion performance of such composites, will occur. Furthermore, graphene exhibits unique optical conductivity due to its strong interaction with light. To reveal how interface effects influence the photothermal conversion performance of these nanocomposites, the optical conductivity of graphene at near-infrared frequencies was introduced to modify the effective medium theory. By combining this with a photothermal conversion model, the photothermal conversion behaviors of GNS–polymer composites are discussed, taking into account the interface effects and optical conductivity characteristics of GNSs.

## 1. Introduction

Due to its superior properties, including near-infrared absorption (~48%), a photothermal conversion efficiency up to ~65 (±5%), and a large surface area (2630 m^2^/g) [1,2,3], graphene is considered an excellent photothermal additive that can be compounded with polymer matrices to form new and smart photothermal nanocomposites [4,5,6]. Under the irradiation of near-infrared (NIR) light, the excellent optical conductivity of such materials, due to the strong light–matter interactions in sp^2^ graphene sheets and high thermal conductivity, results in the efficient conversion of optical energy into thermal energy. This effect causes the temperature of the matrix to increase rapidly. It has been reported that the temperature of GNS–polymer composites with a GNS content of only 5 wt% can rapidly increase by ~55 °C within just 5 s when exposed to NIR [7,8,9]. Due to this excellent photothermal effect, these kinds of smart photothermal nanocomposites have promising applications in a wide variety of fields, such as photothermal therapy, drug delivery, and antimicrobial activity [10,11,12,13]. Additionally, this type of composite can exhibit various mechanical responses, such as shrinkage and bending, under local NIR irradiation, positioning it as a promising candidate for photothermally driven microdevices, including intelligent sensors, wireless actuators, and biomimetic robotics [14]. However, precisely regulating the photothermal conversion behaviors has become one of the major challenges limiting the practical application of these types of nanocomposites.

The photothermal effect of GNS–polymer nanocomposites is mainly determined by their effective electrical conductivity and permittivity. Similarly to other nanocomposites, these properties mainly depend on the composite’s microstructure, including the concentration of GNSs in the matrix, the GNS aspect ratio, and strong interfacial interactions between the polymer and GNSs [15,16]. It has been reported that when the GNS concentration in a polymer matrix exceeds the percolation threshold, the GNSs are likely to self-assemble into a conductive network, storing an electrical charge at their interfaces with the insulating polymer according to the Maxwell–Wagner–Sillars (MWS) principle. In addition, electrical transport in GNS–polymer nanocomposites can occur not only through direct contact between the conductive GNSs but also through electrical tunneling between conductive GNSs that are sufficiently close to one another [17,18,19]. These interface effects are regarded as key factors affecting changes in effective electrical conductivity and permittivity, which are also considered critical to achieving the precise regulation of photothermal conversion in GNS–polymer nanocomposites [20,21,22].

Most existing studies focus primarily on photothermal applications and the use of experimental methods to improve the photothermal effect. In order to determine the mechanisms that regulate photothermal conversion performance in graphene–polymer nanocomposites, Li et al. [23,24,25] successively presented analytical approaches to photothermal conversion based on Maxwell’s electromagnetic wave theory and energy balance relationships. In these models, effective medium theory was used to obtain effective complex permittivity and to determine the mechanism by which the graphene concentration and size, as well as the intensity of the NIR irradiation, influence the photothermal conversion behaviors of such composites. However, these models merely assumed that GNSs are randomly oriented in the polymeric matrix, and the percolation phenomenon was not considered. Therefore, the effects of electrical transport or electrical tunneling on photothermal conversion were not discussed. Furthermore, how varying light frequency influences photothermal conversion remains unclear.

In this paper, a modified photothermal conversion model is proposed that combines the energy balance relation and a modified effective medium theory, taking into account the interface effects and photoconductivity of graphene under NIR irradiation. Based on the new effective medium theory developed by Weng [19], the photoconductivity of graphene is introduced to modify the formation of effective electrical conductivity and dielectric permittivity, whilst also considering the contribution of electron tunneling and MWS polarization. This was then combined with the energy balance equation and Newton’s cooling law to build the photothermal conversion model that is presented in Section 2 in order to describe the process of photothermal conversion in graphene–polymer composites under NIR irradiation. The energy difference between the energy received from the absorption of light and the dissipation of energy into the external environment is defined as the generalized driving force behind the photothermal conversion process in composites under NIR irradiation. In Section 3, the temperature responses of nanocomposites under NIR are simulated to elucidate how interfacial effects influence photothermal energy conversion. The paper is concluded in Section 4 with a general discussion of the photothermal conversion model.

## 2. Analytical Photothermal Conversion Model

**Figure 1:**
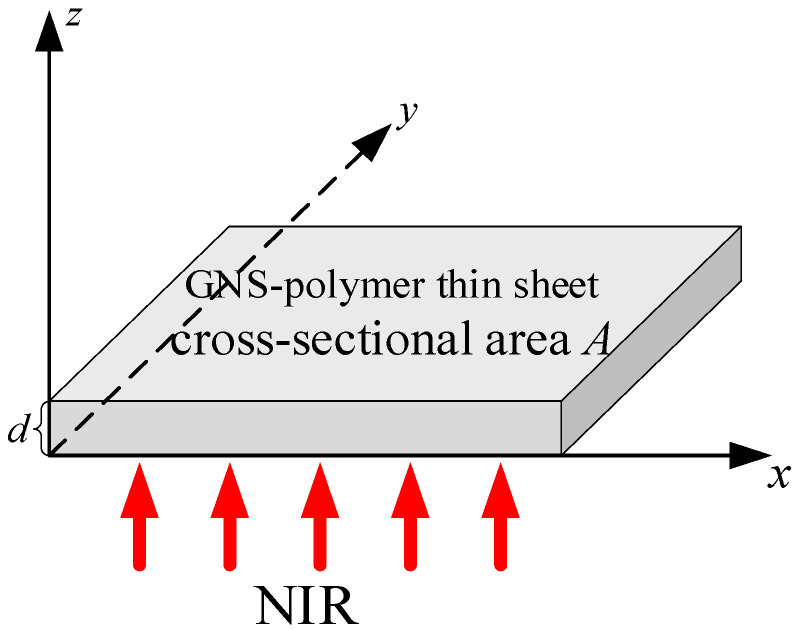
Analytical model of photothermal conversion in graphene–polymer nanocomposite.

As shown in Figure 1, a thin uniform sheet of a GNS–polymer nanocomposite with a cross-sectional area *A* and thickness *d* is proposed to model photothermal conversion behavior under NIR irradiation. Based on the continuum energy balance relationship, the temperature variation in the nanocomposite is mainly governed by a generalized driving force *q*, which is defined as the energy difference between the energy absorbed under NIR irradiation $Q_{I}$ and the energy dissipated due to the temperature difference in the surrounding environment $Q_{ext}$, and this driving force can be expressed as [25,26]

(1) $$\left(m_{p}C_{p}+m_{g}C_{g}\right)\frac{dT\left(t\right)}{dt}=q$$

where *t* is the irradiation time; *T*(*t*) is the temperature of the composite at time *t*; $m_{p}$ and $m_{g}$ are the mass of the polymer matrix and GNSs, respectively; and $C_{p}$ and $C_{g}$ are the specific heat capacity of the polymer matrix and the GNSs, respectively. The energy absorbed due to the surface plasmon resonance of GNSs under NIR irradiation can be described as

(2) $$Q_{I}=I\left(1-e^{-\alpha d}\right)\eta$$

with NIR power *I* and the efficiency of transducing absorbed resonant radiation into thermal energy via plasmons η. This is defined as 65% based on the experimental data [1]. α is the coefficient used to describe the absorbance of the GNS–polymer nanocomposites to NIR light, which is determined by the effective electrical conductivity $\sigma_{e}$ and permittivity $\varepsilon_{e}$ of GNS–polymer nanocomposites and is obtained in Section 3.

Based on Newton’s law of cooling, the dissipated energy is assumed to be linearly proportional to the temperature difference ΔT between the composite and the surrounding environment $T_{0}$. The composite is assumed to be uniform and isotropic. Its heat transfer coefficient *k* is a constant. Then, the rate of energy flowing out of the composite sheet is given by

(3) $$Q_{ext}=kA\Delta T$$

Combining Equations (1)–(3), the variation in the temperature difference under NIR irradiation satisfies the differential Equation (4).

(4) $$\frac{d\Delta T\left(t\right)}{dt}=\frac{I\left(1-e^{-\alpha d}\right)\eta }{m_{p}C_{p}+m_{g}C_{g}}-\frac{kA}{m_{p}C_{p}+m_{g}C_{g}}\Delta T$$

When the source of NIR irradiation is turned off, no more energy is absorbed, and $Q_{I}=0$. The composite begins to cool due to the heat exchange with its surroundings. Therefore, Equation (4) is simplified to

(5) $$\frac{d\Delta T\left(t\right)}{dt}=-\frac{kA}{m_{p}C_{p}+m_{g}C_{g}}\Delta T$$

By solving Equations (4) and (5), the temperatures of the composite before and after NIR irradiation are as follows:

(6) $$T\left(t\right)=\left\{\begin{array}{c} T_{0}+\left(\frac{I\left(1-e^{-\alpha d}\right)\eta }{kA}\right)\left(1-\exp\left(-\frac{kA}{m_{p}C_{p}+m_{g}C_{g}}t\right)\right),\;\left(Q_{I}\neq 0\right)\\ T_{0}+\left(T_{max}-T_{0}\right)\exp\left(-\frac{kA}{m_{p}C_{p}+m_{g}C_{g}}t\right),\;\left(Q_{I}=0\right) \end{array}\right.$$

where $T_{max}$ is the maximum temperature of the composite sheet when the absorbed energy equals the dissipation energy, $Q_{I}=Q_{ext}$. This can be obtained by combining Equations (2) and (3) and is given as follows:

(7) $$T_{max}=T_{0}+\frac{I\left(1-e^{-\alpha d}\right)\eta }{kA}$$

## 3. Determination of Absorption Coefficient

According to Maxwell’s electromagnetic field theory, the electric and magnetic fields (***E*** and ***H***) in GNS–polymer nanocomposites under NIR irradiation satisfy Maxwell’s equations [24,27],

(8) ∇⋅E=0

(9) ∇⋅H=0

(10) $$\nabla \times E=-\frac{\mu }{c}\frac{\partial H}{\partial t}$$

(11) $$\nabla \times H=\frac{\varepsilon_{e}}{c}\frac{\partial E}{\partial t}+\frac{\sigma_{e}}{c}E$$

And the electric field can generally be expressed as

(12) $$E=E_{0}\exp j\left(\hat{q}\cdot r-\omega t\right)$$

In the above equations, ω, $\hat{q}$, and *c* are the angular frequency, wave vector, and vacuum velocity of the NIR radiation, respectively. Due to the weak magnetism of GNSs and the polymer matrix, in this paper it is assumed that the relative equivalent magnetic permeability of GNS–polymer nanocomposites is 1.

Combining Equations (8)–(12), using the wave vector–refractive index relationship *n* and extinction coefficient *κ* given in Equation (13), gives us

(13) $$\hat{q}=\frac{\omega }{c}\left(n+j\kappa \right)$$

where the extinction coefficient *κ* can be obtained and is determined by the effective electrical conductivity $\sigma_{e}$ and the permittivity $\varepsilon_{e}$ of GNS–polymer nanocomposites

(14) $$\kappa =\frac{1}{\sqrt{2}}{\left[{\left({\left(\varepsilon_{e}\right)}^{2}+{\left(\frac{\sigma_{e}}{\omega }\right)}^{2}\right)}^{\frac{1}{2}}-\left(\varepsilon_{e}\right)\right]}^{\frac{1}{2}}$$

Then, according to the relationship between the absorption coefficient and the extinction coefficient, the absorption coefficient α can be expressed as

(15) $$\alpha =\frac{2\omega \kappa }{c}=\frac{\sqrt{2}\omega }{c}{\left[{\left({\left(\varepsilon_{e}\right)}^{2}+{\left(\frac{\sigma_{e}}{\omega }\right)}^{2}\right)}^{\frac{1}{2}}-\left(\varepsilon_{e}\right)\right]}^{\frac{1}{2}}$$

In order to consider the effects of electron tunneling, the Maxwell–Wagner–Sillars (MWS) interface effect, and the loading frequency on the complex effective conductivity ${\hat{\sigma }}_{e}=\sigma_{e}+j\omega \varepsilon_{e}$ of GNS–polymer nanocomposites, the GNSs are assumed to be oblate spheroids coated by a thin interlayer. The coated GNSs, with a complex conductivity ${\hat{\sigma }}_{i}^{c}=\sigma_{i}^{c}+i\omega \varepsilon_{i}^{c},\left(i=1,3\right)$ in their two half-axis directions, are regarded as new fillers in the matrix. Therefore, the complex conductivity of GNS–polymer nanocomposites satisfies the following relationship [19]:

(16) $$\left(1-f\right)\frac{{\hat{\sigma }}_{m}-{\hat{\sigma }}_{e}}{{\hat{\sigma }}_{e}+\left(1/3\right)\left({\hat{\sigma }}_{m}-{\hat{\sigma }}_{e}\right)}+\frac{1}{3}f\left[\frac{2\left({\hat{\sigma }}_{1}^{c}-{\hat{\sigma }}_{e}\right)}{{\hat{\sigma }}_{e}+S_{11}\left({\hat{\sigma }}_{1}^{c}-{\hat{\sigma }}_{e}\right)}+\frac{{\hat{\sigma }}_{3}^{c}-{\hat{\sigma }}_{e}}{{\hat{\sigma }}_{e}+S_{33}\left({\hat{\sigma }}_{3}^{c}-{\hat{\sigma }}_{e}\right)}\right]=0$$

where *f* is the volume fraction of GNSs in the matrix, and ${\hat{\sigma }}_{m}=\sigma_{m}+j\omega \varepsilon_{m}$ is the complex conductivity of the polymer. The conductivity and permittivity of the coated GNSs are determined by the conductivity and permittivity of GNSs ($\sigma_{i}^{g}$ and $\varepsilon_{i}^{g}$) and the interlayer ($\sigma_{int}$ and $\varepsilon_{int}$), which are, respectively, given as

(17) $$\sigma_{i}^{c}=\sigma_{int}\left[1+\frac{\left(1-f_{int}\right)\left(\sigma_{i}^{g}-\sigma_{int}\right)}{f_{int}S_{ii}\left(\sigma_{i}^{g}-\sigma_{int}\right)+\sigma_{int}}\right]$$

(18) $$\varepsilon_{i}^{c}=\varepsilon_{int}\left[1+\frac{\left(1-f_{int}\right)\left(\varepsilon_{i}^{g}-\varepsilon_{int}\right)}{f_{int}S_{ii}\left(\varepsilon_{i}^{g}-\varepsilon_{int}\right)+\varepsilon_{int}}\right]$$

The components of the depolarization tensor ***S*** are related to the aspect ratio (the ratio of the thickness to the diameter of oblate spheroids, ξ≪1) of GNSs and are defined as

(19) $$S_{11}=S_{22}=\frac{\xi }{2{\left(1-\xi^{2}\right)}^{3/2}}\left[\arccos\xi -\xi {\left(1-\xi^{2}\right)}^{1/2}\right],S_{33}=1-2S_{11}$$

where $f_{int}$ is the volume fraction of the interlayer in the coated GNS filler, is related to the thickness of interlayer *h* and the GNSs λ, and is given by

(20) $$f_{int}=\frac{\lambda }{2}{\left(\frac{\lambda }{2\xi }\right)}^{2}/\left(\frac{\lambda }{2}+h\right){\left(\frac{\lambda }{2\xi }+h\right)}^{2}$$

To reflect the influence of the frequency-dependent electron tunneling on the effective conductivity and permittivity, Cauchy’s statistical function *F* and Dyre’s hopping function *p* are introduced [17] as follows:

(21) $$F\left(f,f^{*},\gamma \right)=\frac{1}{\pi }\arctan\left(\frac{f-f^{*}}{\gamma }\right)+\frac{1}{2}$$

(22) $$p\left(\omega \right)=\frac{\omega t_{\sigma }{\left(\arctan(\omega t_{\sigma })\right)}^{2}}{{\left[0.5\mathrm{Ln}\left(1+{\left(\omega t_{\sigma }\right)}^{2}\right)\right]}^{2}+{\left(\arctan(\omega t_{\sigma })\right)}^{2}}$$

with a common resistance-like function

(23) $$\varphi \left(f,f^{*},\gamma \right)=\frac{F\left(1,f^{*},\gamma \right)-F\left(f,f^{*},\gamma \right)}{F\left(1,f^{*},\gamma \right)-F\left(0,f^{*},\gamma \right)}$$

and the electrical conductivity and dielectric permittivity of the interlayer can, respectively, be expressed as

(24) $$\sigma_{int}=\sigma_{int}^{0}p\left(\omega \right)/\varphi \left(f,f^{*},\gamma_{s}^{\sigma }\right)$$

(25) $$\varepsilon_{int}=\frac{\varepsilon_{int}^{0}}{\kappa \left(f,f^{*},\gamma_{inf}^{\varepsilon }\right)}+\frac{\varepsilon_{int}^{0}/\varphi \left(f,f^{*},\gamma_{s}^{\varepsilon }\right)-\varepsilon_{int}^{0}/\varphi \left(f,f^{*},\gamma_{inf}^{\varepsilon }\right)}{1+\omega^{2}\tau_{\varepsilon }^{2}}$$

In the above equations, γ is a scale parameter characterizing the rise in electron tunneling and the formation of nanocapacitors at the interface as the GNS concentration passes through the percolation threshold $f^{*}=9S_{33}\left(1-S_{33}\right)/\left(-9S_{33}^{2}+15S_{33}+2\right)$. $\gamma_{s}^{\sigma }$,$\gamma_{s}^{\varepsilon }$, and $\gamma_{inf}^{\varepsilon }$ describe, respectively, the parameter γ independent of the frequency and at infinite frequency; $t_{\sigma }$ is the characteristic time of the electron tunneling; $\tau_{\varepsilon }$ is the relaxation time; and $\sigma_{int}^{0}$ and $\varepsilon_{int}^{0}$ are the original conductivity and permittivity of the interlayer at f→0.

Furthermore, frequency is also an important factor for the conductivity of GNSs. It has been reported that graphene exhibits a universal optical conductivity under IR irradiation, mainly resulting from the intraband transition of photons or free carriers. Then, based on the Drude model, the complex bulk conductivity of the graphene conforms to the following expression [28]:

(26) $$\sigma^{g}(\omega )=\frac{iD}{\pi (\omega +i\tau^{-1})}$$

with the Drude weight (*D*) depending on the thickness of the GNSs. By assuming that each layer of the GNSs has the same Fermi energy level, mobility, and relaxation time, the Drude weight (*D*) can be described by

(27) $$D=\frac{e^{2}E_{f}}{{\left(\hbar /2\pi \right)}^{2}}$$

where *e* is the electron charge, $E_{f}$ is the Fermi energy level, and ħ is the reduced Planck’s constant. τ is the electron relaxation time expressed as $\tau =\upsilon E_{f}/ev_{f}^{2}$ with the Fermi velocity $v_{f}$ and carrier mobility υ. To describe the anisotropic electrical conductivities of GNSs in the in-plane and out-of-plane directions, the thickness $\delta_{i}$ and cross-sectional area $\chi_{i}$ in different directions are introduced to modify the Drude model; then, the electrical conductivity and permittivity of the GNSs are, respectively, described as [29,30]

(28) $$\sigma_{i}^{g}=\frac{D\tau^{-1}}{\pi \left(\omega^{2}+\tau^{-2}\right)}\frac{\delta_{i}}{\chi_{i}}$$

(29) $$\varepsilon_{i}^{g}=\frac{D}{\pi \left(\omega^{2}+\tau^{-2}\right)}\frac{\delta_{i}}{\chi_{i}}$$

## 4. Results and Discussion

In this paper, the effects of the GNS concentration, aspect ratio, and frequency on the photothermal conversion behaviors of GNS–polymer nanocomposites under NIR irradiation are determined based on the modified photothermal conversion model and by taking into account the interface effects and photoconductivity of the GNSs. Firstly, the influence of interface effects and frequency on the conductivity and permittivity are discussed based on the material parameters shown in Table 1 [17,31].

**Figure 2:**
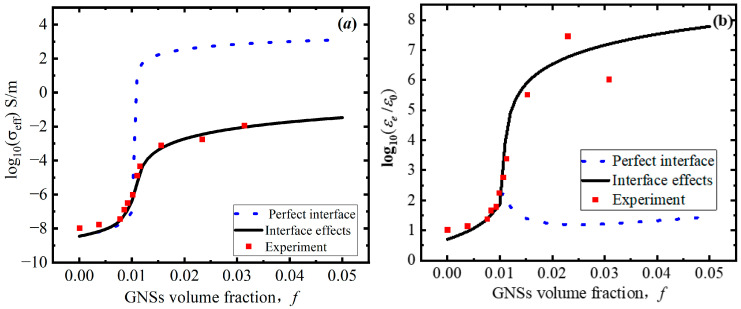
Effect of interface effects on equivalent properties of GNS–polymer nanocomposite as GNS volume fraction increases: (**a**) equivalent conductivity and (**b**) equivalent permittivity.

As shown in Figure 2, the calculated equivalent conductivity and permittivity, taking into account interface effects and graphene photoconductivity, agree well with the experimental data [31] at a frequency of 1000 Hz. In contrast, the results obtained under the assumption of a perfect interface show significant deviation. The equivalent conductivity and permittivity of the GNS–polymer nanocomposite are observed to increase with the increasing GNS volume fraction until the percolation threshold is reached. This is due to the negligible MWS effect caused by the large distance between individual GNSs. The equivalent properties are mainly determined by the properties of the GNSs and the frequency. Therefore, the tendency to increase with the increasing volume fraction is the same whether the interface is assumed to be perfect or imperfect. When the volume fraction is equal to the percolation threshold, the GNSs are arranged in a parallel orientation for self-assembly into a conductive network due to the MWS effect. The effective conductivity of the nanocomposite is mainly determined by interface tunneling, which leads to the conductivity of an imperfect interface being lower than that of a perfect interface, as shown in Figure 2a. Meanwhile, the conductive networks cause significant electron accumulation at the interface, which leads to a rapid increase in the equivalent permittivity, as shown in Figure 2b.

**Figure 3:**
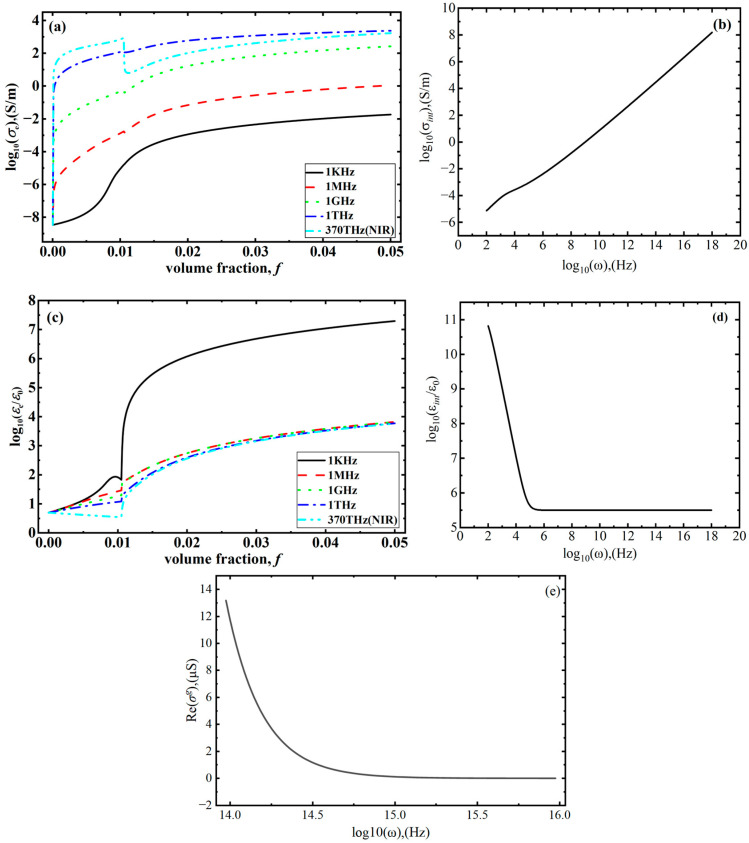
The effect of frequency on the effective properties of the GNS–polymer nanocomposite: (**a**) effective conductivity; (**b**) interfacial conductivity; (**c**) effective permittivity; (**d**) interfacial permittivity; and (**e**) graphene photoconductivity.

In this section, we continue to explore the effects of the GNS concentration and frequency on the effective conductivity and permittivity. As shown in Figure 3a, the effective conductivity of the GNS–polymer composite increases with the increasing volume fraction of GNSs whether the frequency is 1 KHz or 1 MHz. This is because the influence of frequency on the MWS effect is relatively weak, the concentration determines the structure of graphene at low frequencies, and a conductive network is formed as the volume fraction reaches the percolation threshold. When nanocapacitors are formed and electrons accumulate at the interface, the effective conductivity and permittivity reach saturation. When the frequency is greater than 1 GHz, the MWS polarization is mainly determined by the frequency, and more electrons will cross the GNSs–polymer interface due to the increasing frequency, as shown in Equation (22), resulting in the conductivity of the interface increasing rapidly, as shown in Figure 3b, while the permittivity decreases, as shown in Figure 3d. This leads to the effective conductivity increasing rapidly at low concentrations, while the dielectric performance shows a downward trend, as shown in Figure 3c. As a conductive network is formed, the effective conductivity and permittivity approach a saturation state. Consequently, frequency-dependent interfacial conductivity and permittivity become the dominant factors governing the effective conductivity and permittivity of the composite at high frequencies. When the frequency extends into the NIR range, the equivalent conductivity decreases rapidly at the percolation threshold and eventually saturates. This behavior arises due to the strong light–matter interactions at NIR frequencies; the excitation frequency approaches the order of the Drude weight and scattering width that govern graphene’s photoconductivity in Equation (26), leading to a rapid decline in photoconductivity with an increasing frequency. Although a conductive network forms at the percolation threshold, the increase in interfacial conductivity driven by the MWS effect cannot compensate for the sharp decrease in the graphene photoconductivity, resulting in a reduction in effective conductivity.

In order to determine how interface effects and frequency affect photothermal conversion behaviors, the temperature response of the photothermal conversion, with and without interface effects, is determined and compared with experimental data for the GNS–PDMS (*Polydimethylsiloxane*) composite [32] and the GNS–PAM (*Poly acrylamide*) composite [33]. The numerical analysis is conducted at a wavelength of 808 nm, a GNS density of 2200 kg m^−3^, a GNS specific heat capacity of 710 J/(kg·K), a PDMS density of 1000 kg m^−3^, a PDMS specific heat capacity of 3500 J/(kg·K), a PDMS heat transfer coefficient of 370 W/(m^2^·K), a PAM density of 1302 kg m^−3^, a PAM specific heat capacity of 3100 J/(kg·K), and a PAM heat transfer coefficient of PAM 95 W/(m^2^·K) [32,33].

**Figure 4:**
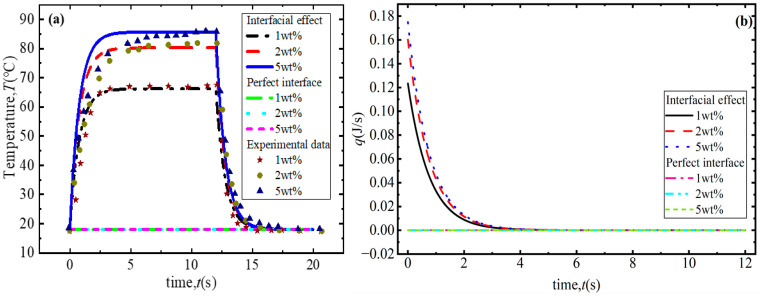

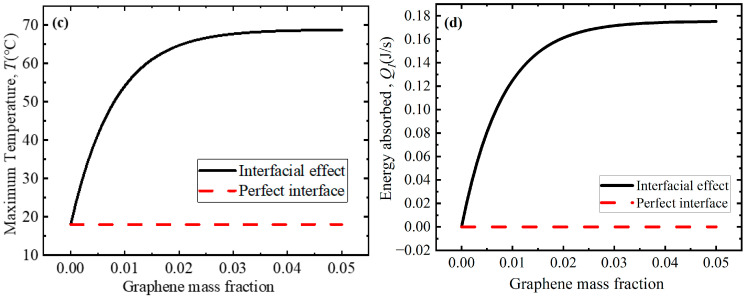
Influence of graphene mass concentration on the photothermal conversion behaviors of the GNS–PDMS composite: (**a**) temperature changes with irradiation time; (**b**) changes in generalized driving force with irradiation time; (**c**) maximum temperature by graphene mass concentration; and (**d**) the absorbed energy by graphene mass concentration.

As shown in Figure 4a, compared to the results for a perfect interface, the numerical results obtained with interface effects taken into account agree well with the experimental results [32]. Under NIR irradiation, the temperature response can be divided into three stages. Firstly, the temperature of the composite increases rapidly as the NIR irradiation begins. This is because frequency-dependent MWS effects induce electrons to cross the GNS–polymer interface, which causes the conductivity to increase rapidly, as shown in Figure 3a. Based on Equation (15), the absorption coefficient, mainly determined by effective conductivity, also increases under these conditions. In addition, due to the strong light–matter interactions, the plasmonic resonance dips of GNSs are not highly pronounced when the photoconductivity is in a state of saturation due to the increase in frequency, as shown in Figure 3e. Therefore, GNS–polymer composites are able to absorb a much greater amount of energy, resulting in the absorbed energy being far greater than the dissipated energy, as shown in Figure 4b. The greater driving force causes the temperature of the composite to rise rapidly. Moreover, with the increase in the irradiation time, the temperature difference between the composite and the surrounding environment gradually increases, resulting in an increase in dissipated energy, which gradually approaches the energy absorbed by the composite, and thus the temperature change gradually decreases. As near-infrared light irradiation continues, the composite material reaches a balance between energy dissipation and absorption, meaning that the energy corresponding to its temperature change satisfies $Q_{I}-Q_{ext}=0$, as shown in Figure 4b. At this point, the temperature of the composite reaches its maximum, corresponding to the temperature saturation stage in Figure 4a, namely the second stage of the photothermal conversion process. At this stage, the maximum temperature is observed to increase with the increase in the GNS concentration shown in Figure 4c. This is due to the number of electron tunneling paths between GNSs increasing with the increase in the GNS concentration, and the number of nanocapacitors increases accordingly. Therefore, the conductivities of the interface and composites clearly increase with the increase in the graphene concentration shown in Figure 3, which causes the absorbed energy to increase due to the increasing dielectric loss shown in Figure 4d. Finally, when NIR irradiation ceases, the composite material no longer experiences energy absorption, with only energy dissipation taking place due to the temperature difference between the composite and the surroundings. As a result, the temperature of the composite gradually decreases until it is consistent with the ambient temperature, as shown in Figure 4a. However, under conditions of a perfect interface, without taking into account MWS polarization and the effect electrons crossing the interface has on effective conductivity, the frequency-dependent GNS photoconductivity decreases rapidly, and the effective conductivity (itself mainly determined by GNS photoconductivity) also decreases and approaches 0. Therefore, under these conditions, the absorbed energy is much lower than for an imperfect interface. The generalized driving force for the temperature change is very small, which leads to only minor temperature changes during the photothermal conversion process. The corresponding maximum temperature is also much lower than that for the imperfect interface.

**Figure 5:**
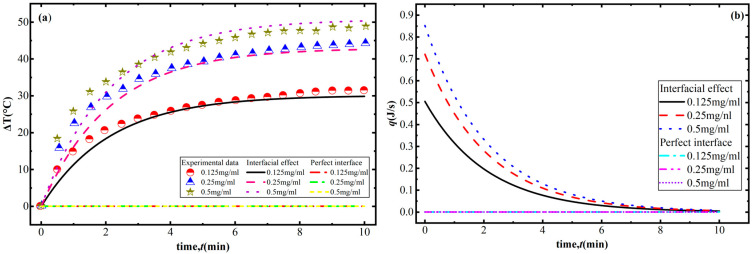
Photothermal conversion behaviors of GNS–PAM composite considering interfacial effects: (**a**) temperature changes with irradiation time and (**b**) changes in generalized driving force with irradiation time.

In order to further verify the developed theory, after considering interfacial effects on the photothermal conversion behaviors of GNS–polymer composites, the numerical analyses for the GNS–PAM composites are also studied. As shown in Figure 5a, across all mass concentrations, a reasonable agreement is observed between our numerical results and the experimental data [33]. However, the temperature increases more slowly compared to the GNS–PDMS composite due to the lower GNS concentration. A decrease in concentration leads to reductions in frequency-dependent MWS-induced electron hopping and electron tunneling paths. As the interfacial conductivity decreases, the conductivity of the composite decreases at the same time. The absorbed energy per unit time is also lower than that of the GNS–PDMS composite. The irradiation time consequently needs to be much longer to make the energy absorbed by the GNS–PAM composite equal the dissipated energy—that is, to cause the driving force to approach zero, as shown in Figure 5b.

**Figure 6:**
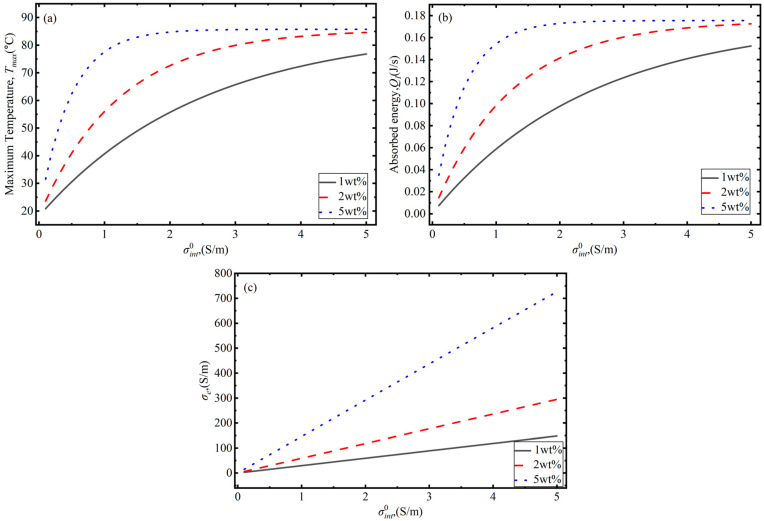
Influence of initial interface conductivity on the photothermal conversion behavior of GNS–PDMS composites considering interface effects: (**a**) maximum temperature; (**b**) absorbed energy; and (**c**) effective conductivity of composite.

To further examine the effect of interface conductivity $\sigma_{int}^{0}$ on the photothermal conversion, how $\sigma_{int}^{0}$ affects the maximum temperature, absorbed energy, and effective conductivity of the composite is depicted in Figure 4a–c. It can be seen from Figure 6b that as the graphene concentration increases (from 1 wt% to 5 wt%), the efficiency of the composite’s energy absorption capacity significantly improves due to the increase in electron tunneling paths. At a GNS concentration of 2 wt%, the energy absorbed by the composite reaches saturation as the initial interface conductivity is approximately 5 S/m. At a concentration of 5 wt%, the absorbed energy reaches saturation at an initial interface conductivity of only 2 S/m. This is because the equivalent conductivity of the composite is in a linear relationship with the initial conductivity, as shown in Figure 6c and Equation (24). Additionally, based on Equations (2) and (15), the absorption coefficient also increases with the increase in effective conductivity, which leads to the exponential function of the absorbed energy decreasing and approaching zero. At the same time, the absorbed energy reaches its maximum, and the corresponding maximum temperature reaches the saturation state, as shown in Figure 6a.

**Figure 7:**
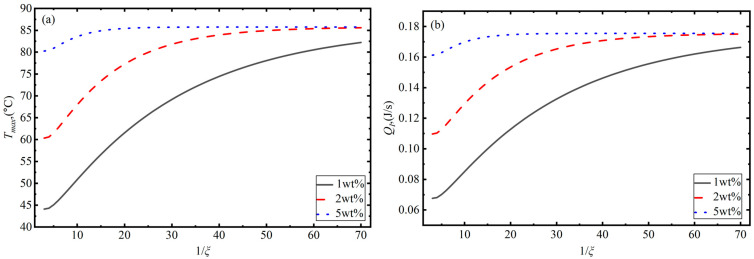
Influence of GNS aspect ratio on the photothermal conversion behavior of GNS—PDMS composites considering interface effects: (**a**) maximum temperature and (**b**) absorbed energy.

In addition to the mass fraction, the aspect ratio of the GNSs is also a key factor influencing the photothermal conversion behaviors of GNS–polymer nanocomposites. The effects of the aspect ratio on the maximum temperature and energy absorption, for a composite exposed to an NIR laser with a power of 0.27 W, are depicted in Figure 7a,b. As shown in Figure 7a, the maximum temperature increases with the GNS concentration, which is the same process as depicted in Figure 4c and is due to the increase in electron tunneling paths improving the material’s dielectric loss capability. In addition, the maximum temperature increases as the aspect ratio decreases. Based on Equations (21)–(24), the decrease in the GNS aspect ratio is observed to result in a lower percolation threshold. However, the resistance-like function exhibits little influence. Therefore, the interface conductivity mainly depends on the frequency. If we assume that the thickness of the GNSs is constant, a smaller aspect ratio will lead to an increase in the lateral dimension of the GNSs, resulting in the increase in the contact area between the GNSs and the matrix strengthening the interface interaction. This will, in turn, reduce interfacial resistivity and increase the equivalent conductivity of the composite. Under these conditions, a much greater quantity of energy will be absorbed by the composite and converted to heat for an equivalent irradiation time, as shown in Figure 7b. Furthermore, the numerical results in Figure 7b indicate that when the graphene aspect ratio reaches a certain threshold, the absorbed energy approaches saturation. Correspondingly, the maximum temperature of the composite under NIR irradiation also reaches saturation, as shown in Figure 7a. This behavior can be attributed to the fact that as the aspect ratio decreases, the surface area of oblate spheroid GNSs expands. This may lead to GNSs coming into contact with each other, forming a conductive network, which results in the conductivity of the composite increasing. Eventually, this causes the absorption coefficient to keep increasing and the absorbed energy per unit time to also increase. When the transmitted light intensity approaches 0, the energy absorbed by the composite reaches its maximum, which means that the maximum temperature tends to be consistent as the aspect ratio decreases under different GNS concentrations.

**Figure 8:**
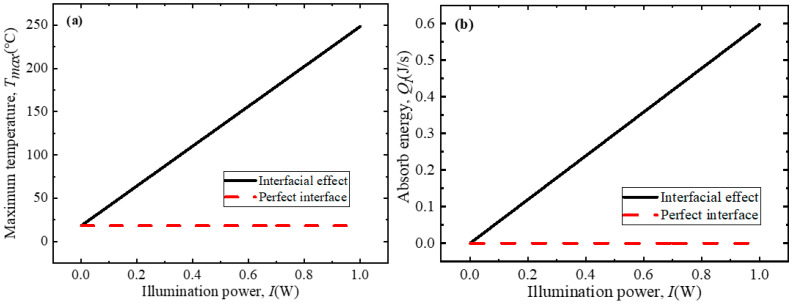
Influence of light intensity on photothermal conversion behavior of GNS–PDMS composite: (**a**) maximum temperature and (**b**) absorbed energy.

A number of studies have shown that the irradiation intensity of the NIR laser also plays an important role in photothermal conversion, and, to elucidate this relationship, the influences of the light intensity on the maximum temperature and energy absorption of composites under perfect and imperfect interface conditions at a mass fraction of 5 wt% were determined and are shown in Figure 8. The numerical results in Figure 8a indicate that the maximum temperature of the composite with interface effects taken into account is much greater than that for a perfect interface. Also, the maximum temperature increases with an increasing irradiation intensity. Notably, the maximum temperature exhibits a linear correlation with the light intensity, a trend that can be attributed to the energy absorption mechanism depicted in Figure 8b; with a constant graphene mass fraction, the energy absorbed per unit time by the composite increases linearly with the irradiation intensity, leading to a corresponding linear increase in the maximum temperature. In contrast, under the assumption of a perfect interface, the absorbed energy exhibits only minor changes, which induces only minor changes to the maximum temperature.

**Figure 9:**
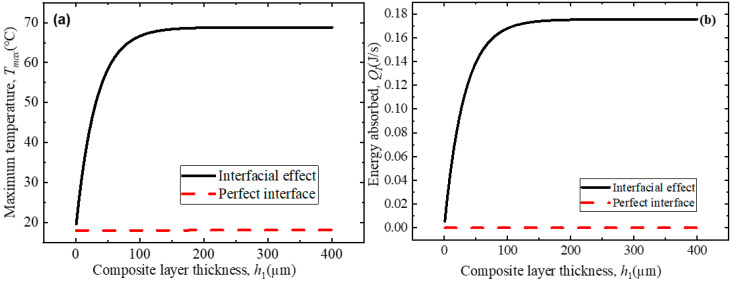
Influence of light irradiation path length on photothermal conversion behavior of GNS–PDMS composite: (**a**) maximum temperature and (**b**) the absorbed energy.

Finally, the influence of the light irradiation path length (the thickness of the composite sheet) on the maximum temperature and energy absorption during photothermal conversion is analyzed for different interface conditions, as shown in Figure 9. As the irradiation path length increases, the energy that can be absorbed by the composite increases significantly, as shown in Figure 9b. When the thickness is greater than 100 μm, the absorbed energy reaches its maximum, no energy is transmitted through the composite sheet, and the maximum temperature reaches saturation and only increases with the increase in the GNS concentration, as shown in Figure 9a. This is because the presence of GNSs and interfaces causes multiple light scattering and reflection pathways inside the medium as light propagates through the composite sheet, resulting in the absorption of light energy by the composite. As the thickness of the composite increases, the light waves that have not yet propagated to the lower surface of the composite sheet are absorbed and converted into thermal energy. Under the assumption of a perfect interface, though the GNSs continue to cause the scattering and reflection of light inside the medium. Based on the above, the effective conductivity will decrease with the NIR frequency. Thus, the absorbed energy exhibits minor changes and consequently induces only minor changes to the maximum temperature.

## 5. Conclusions

To describe the photothermal conversion performance of graphene–polymer nanocomposites under NIR irradiation, we devised a modified photothermal conversion model based on the effective medium theory, Maxwell’s electromagnetic wave theory, and the energy balance equation. This paper discusses the effects of interfacial phenomena between the graphene and the polymer matrix, as well as the photoconductivity characteristics of graphene experiencing temperature changes under NIR irradiation. Additionally, the effects of the light radiation intensity and graphene size on photothermal conversion are incorporated. The findings elucidate the mechanisms by which interfacial characteristics influence the equivalent dielectric constant and conductivity of the composites, as well as their role in the photothermal conversion behavior of such materials. The results indicate that incorporating interfacial effects and the frequency-dependent graphene photoconductivity enables a more accurate characterization of the photothermal conversion behavior and response mechanisms of graphene–polymer nanocomposites under NIR irradiation.

## Figures and Tables

**Table 1:** Physical parameters used in the analysis of photothermal conversion [17,31].

Physical Parameters Used in the Calculation	Values
Electrical conductivity of polymer matrix, $\sigma_{m}(S/m)$	3.5 × 10^−9^
Relative dielectric permittivity of polymer matrix, $\varepsilon_{m}/\varepsilon_{0}$	5
Electric conductivity of the interface at f=0, $\sigma_{int}^{0}(S/m)$	1.5 × 10^−5^
Relative dielectric permittivity of the interface at f=0, $\varepsilon_{int}^{0}/\varepsilon_{0}$	3
Scale parameter of electron tunneling at the interface, $\gamma_{s}^{\sigma }$	0.002
Scale parameter of the formation of nanocapacitors in the static state, $\gamma_{s}^{\varepsilon }$	2.8 × 10^−13^
Scale parameter of the formation of nanocapacitors at the infinite frequency, $\gamma_{inf}^{\varepsilon }$	1.5 × 10^−7^
Characteristic time of electron tunneling, $t_{\sigma }$ (s)	1 × 10^−4^
Relaxation time of Debye theory, $\tau_{\varepsilon }$ (s)	2 × 10^−3^
Fermi energy level for GNSs, $E_{f}$ (eV)	0.6
Carrier mobility for GNSs, υ (m^2^/(Vs))	1
Fermi velocity for GNSs, $v_{f}$ (m/s)	1 × 10^6^

## Data Availability

The original contributions presented in this study are included in the article. Further inquiries can be directed to the corresponding author.
